# Report of a Case of the Difficulties Met with in Primiparous Women

**Published:** 1893-08

**Authors:** A. E. Garrett

**Affiliations:** Sulphur Springs, Texas


For Texas Medical Journal.

### REPORT OF A CASE OF THE DIFFICULTIES MET WITH IN PRIMIPAROUS WOMEN.

BY A. E. GARRETT, M. D., SULPHUR SPRINGS, TEXAS.

ON September 2, 1892, I was called to see Mrs. E. K., who was suffering from a slight rise of temperature, nausea, vomiting, and complaining of severe cramping pains in the lower part of the abdomen. She was at the time "unwell." From her I learned that at each menstrual period she suffered as only women who have severe dysmenorrhœa can. I gave her, as is usual in those cases, opiates to relieve the pain, and put her on tonic treatment, together with dioviburnia, with instruction if this and the dioviburnia did not lessen the pain at the next period, I would then examine the uterus and see what was to be done. When the next period came she missed; and then suspecting pregnancy (which afterwards proved to be the case), I decided not to examine the uterus. From that time until January 20th she went without an untoward symptom. Up and going when, and where she chose. On January 20th, she began to be threatened with miscarriage. I enjoined perfect quietude and gave opiates. She got on as well as any one could under similar circumstances. On February 13th I was again called and found that on the day previous she had been buggy riding, and was now conplaining with severe pains in the lower part of the abdomen, restless, nervous, tossing from one side of the bed to the other; first one position and then another, not occupying the same position exceeding fifteen seconds at a time. I made a digital examination, and found the os not at all dilated and cervix hard, and cartilaginous. Notwithstanding this, when the pains would come up, which they would do at intervals of about every five minutes, I could feel the contractions of the uterus very perceptibly. I gave her a hypodermic injection of morphia sulph. grain ¼, and atrop. sulph. grain 1-150 and waited. In one hour she was no better, and I repeated the morphia, grain ¼, and in one hour more I gave her ten grains of chloral hydrate, and 30 grains of potass. brom. Then in one hour more repeated the morphia. After repeating this every hour for four hours, she sank into a restless, troubled sleep, from which she would wake with a start and nearly jump out of the bed at the least noise or unusual sound. (For instance the striking of a clock would so alarm her that we would have to hold her on the bed.)

On the next day she was considerably improved, and after a day or two was again up, and going any and every where. Contrary to my advice she would get in a buggy and go riding every day. Again on April 2d I was called and found her as before with the exception at this time, the os was somewhat dilated, soft and patulous, and if anything, the pains were greater than before. This time began on morphia ½ grain doses hypodermically. At 8 p. m., I gave her ½ grain of morphia hypodermically. At 8:30 p. m., 20 grains of chloral hydrate; then at 9:30 p. m., ½ grain of morphia. At 10:30 p. m., 20 grains of chloral hydrate, and 30 grains of potass. brom. Then at 12:30 a. m., I gave her ¼ grain of morphia, and 20 grains chloral hydrate, and she again, in about one hour began this sleep, which seemed to be more from sheer exhaustion than from soporifics; and again she would awake at almost any unusual sound, and require the combined strength of two or three persons to hold her on the bed. I left her at 3 a. m. Called again next day and found her much improved from the condition on the night previous. Pulse 125 per minute, temperature 99¾, but complaining of no pain. On the night of the 4th and fifth instant, this same thing was to go through with. On the night of the 5th I concluded to try MacMunn's elixir of opium, to see if it would quiet her. I accordingly began with teaspoonful doses at 9 p. m., and repeated them every hour. By 1 p. m., or within four hours I had given her an entire bottle of MacMunn's Elixir of Opium, and the only effect I could see was the contraction of the pupils. No slowing of the respiration, no soporific effect whatever. I then gave 30 grains chloral hydrate, and again left her. The next day, as usual, she was somewhat better, or at least she could sleep as much as two hours a good quiet sleep. She improved slowly, and again got up. She again began buggy riding and visiting, going as she chose and where she chose, so again on April 23rd, after being out all day in the buggy with her husband, she had me call. I found her with an exactly similar attack to what she had been having all along. I gave her ¾ grains of morphia hypodermically, and introduced a speculum, and cauterized the cervix, both inside and outside with pure carbolic acid. Somewhat to my surprise, she went off into a quiet and peaceful sleep, and rested all night. Was refreshed the next morning and said she felt better than for a month. Again she got up, and was going over town visiting. On May 8th I was again sent for. Finding her as usual, I gave her ¾ grain of morphia hypodermically, and in one hour a grain of phosphate codeine. In one hour again ¾ grain of morphia, and in one hour ¾ grain of codeine. This was repeated alternately for six hours with no visible effect whatever. On the night of the 9th she went through with the same thing. Again on the 10th I used carbolic cauterization in connection with the opiates, and this time got no effect from either. From this on until the 18th of May, I run through the whole category of soporifics, finding none that had any effect upon her. On the 21st of May I was called again, and this time finding the os perfectly dilatable I had Doctor E. P. Becton called, and we discussed the advisability of dilating the womb, and delivering her. But since she was in no immediate danger, we concluded to try her on 40 grains of chloral hydrate per rectum every two hours until she had taken four doses, or until sleep was produced. I went to the drug store and had this prepared for use. Now she had a solution of chloral at the house of 80 grains per ounce, and she had taken from two to three teaspoonful doses. I came back and gave her 40 grains of chloral hydrate of the mixture I had prepared for use by the rectum, and left instructions for her husband to repeat it every two hours until she went to sleep. Being anxious to go to sleep, and not knowing what this was that I gave her by the rectum, she had her husband to give her a tablespoonful of the solution by the mouth, which she had there, which was 80 grains of chloral hydrate, she took every three hours until she had taken three doses. The next morning when I came and asked her how she felt, she said, "Doctor, I wanted to sleep last night, and as you left me I had my husband to give me a tablespoonful of that chloral mixture I had here every time he gave me the other medicine by enema, and I got about two hours sleep!" On May 28th I delivered her of a healthy living boy. She had a normal labor in every respect, and made as good recovery as I ever saw any one make. She is 19 years old. This is her first pregnancy, having been married about two years. Never took a dose of morphine or other narcotic until I gave it to her last September, and has never taken any since except what I prescribed. This sounds like one of Baron Munchausen's tales, but every word is true, and can be substantiated. My object in reporting this case, is to call the attention of the Association to two points.

First. The enormous quantities of morphine, chloral, opium, codeine, and other narcotics taken without producing any visible effect whatever, when taken either by the mouth, by the rectum, or hypodermically.

Second. The tolerance of the uterus, its rough handling, its cauterization, not only of the os, but the cervix, and cervical canal, with complete arrest of all pains in the first instance, instead of causing abortion; and in the second, without any effect whatever. Now I know that the terminal filaments of the nerves surrounding the os were probably in a hyperæsthetic condition, and the cessation of pain in the first instance, was probably due to the anæsthetic effect of the local application of the carbolic acid on these nerve terminals. But why in the second case, if this pain was due either to central or peripheral irritation, was it not controlled on the one hand by the enormous quanties of narcotics taken, or on the other by the application of the local anæsthetic to the nerve terminals?

